# Auditory Compensation for Head Rotation Is Incomplete

**DOI:** 10.1037/xhp0000321

**Published:** 2016-11-14

**Authors:** Tom C. A. Freeman, John F. Culling, Michael A. Akeroyd, W. Owen Brimijoin

**Affiliations:** 1 School of Psychology, Cardiff University; 2 Medical Research Council Institute of Hearing Research, University of Nottingham; 3 Medical Research Council/Chief Scientist Office Institute of Hearing Research—Scottish Section, Glasgow Royal Infirmary

**Keywords:** hearing, motion perception, head rotation

## Abstract

Hearing is confronted by a similar problem to vision when the observer moves. The image motion that is created remains ambiguous until the observer knows the velocity of eye and/or head. One way the visual system solves this problem is to use motor commands, proprioception, and vestibular information. These “extraretinal signals” compensate for self-movement, converting image motion into head-centered coordinates, although not always perfectly. We investigated whether the auditory system also transforms coordinates by examining the degree of compensation for head rotation when judging a moving sound. Real-time recordings of head motion were used to change the “movement gain” relating head movement to source movement across a loudspeaker array. We then determined psychophysically the gain that corresponded to a perceptually stationary source. Experiment 1 showed that the gain was small and positive for a wide range of trained head speeds. Hence, listeners perceived a stationary source as moving slightly opposite to the head rotation, in much the same way that observers see stationary visual objects move against a smooth pursuit eye movement. Experiment 2 showed the degree of compensation remained the same for sounds presented at different azimuths, although the precision of performance declined when the sound was eccentric. We discuss two possible explanations for incomplete compensation, one based on differences in the accuracy of signals encoding image motion and self-movement and one concerning statistical optimization that sacrifices accuracy for precision. We then consider the degree to which such explanations can be applied to auditory motion perception in moving listeners.

## Public Significance Statement

Rotating the head in front of a static sound creates the same acoustic information as a sound that moves around the head. Surprisingly little is known about how hearing resolves simple ambiguities like this, despite the fact that our heads often move in normal everyday listening. If the auditory system failed to compensate for head movement, it would mean that the acoustic world would be a continual and bewildering combination of the movement of sound sources and our own movement. To get at the question of how we compensate for self-motion, we used a special technique in which sounds moved across an array of speakers whenever the listener made a head movement. We found that compensation was quite good but not complete. Critically, we found that listeners judged sounds that moved slightly with them to be more stationary than those that were actually still. We speculate on the different ways that self-movement compensation could be achieved and why our listeners made small mistakes.

Hearing and vision are confronted by a similar problem when the perceiver moves: Actions like rotating the head cause the sensory apparatus to shift with respect to the scene. In vision, this creates movement in the image on the retina. In hearing, it creates smooth changes in sound localization cues. We refer to both these effects as “image motion.” Until the observer knows certain characteristics of his or her own self-movement like velocity, image motion remains ambiguous. One way the visual system solves this type of problem is to use various combinations of motor commands, proprioception, and vestibular information to estimate eye and head velocity (Angelaki & Hess, 2005; Crowell, Banks, Shenoy, & Andersen, 1998; Freeman, Champion, & Warren, 2010; Furman & Gur, 2012; von Holst, 1954). These are known collectively as “extraretinal signals” because their origin is from a source other than the retina; they therefore serve as a reference for the image motion created by self-movement. Here we investigate whether the auditory system uses a similar strategy when estimating the movement of sound sources during head rotation. Specifically, we asked whether the auditory system uses “extracochlear signals” when interpreting acoustic image motion, in keeping with the definition and use of “extraretinal” signals known to accompany smooth pursuit eye movements in vision.

Unlike vision, the encoding of acoustic image motion is made difficult by the fact that auditory cues to location are more implicit than those found in vision. The images of auditory objects do not lie on a convenient array of spatially contiguous detectors, and so location must be reconstructed using binaural cues based on timing and intensity differences across the ears, as well as monaural cues based on position-dependent spectral changes created by the filtering properties of the pinna (Middlebrooks & Green, 1991). Information about acoustic image motion is then carried by dynamic changes in these cues (e.g., Zakarauskas & Cynader, 1991). Given the cues’ implicit nature, it is perhaps unsurprising to find little evidence for velocity-tuned motion units like those known to exist early on in the visual pathway. As a consequence, evidence for an auditory motion aftereffect is not compelling (Grantham, 1989; Neelon & Jenison, 2003), and properties such as speed are not preferred by listeners over the associated cues of displacement, duration, and location (Carlile & Best, 2002; Freeman et al., 2014). The latter finding is in stark contrast to vision, where speed typically dominates (Champion & Freeman, 2010; Reisbeck & Gegenfurtner, 1999; Wardle & Alais, 2013). Nevertheless, the auditory system is still able to use velocity information to discriminate movement when displacement, duration, and location are made unreliable (Carlile & Best, 2002; Freeman et al., 2014). Moreover, above threshold, listeners are able to track moving sounds with their heads (Leung, Wei, Burgess, & Carlile, 2015) and can also judge time-to-impact for motion along the azimuth as accurately as vision (Wuerger, Meyer, Hofbauer, Zetzsche, & Schill, 2010). There is some evidence that there exist areas in the auditory cortex sensitive to dynamic changes in location cues (Altman, 1968; Jenison, Schnupp, Reale, & Brugge, 2001; Jiang, Lepore, Poirier, & Guillemot, 2000; Sovijärvi & Hyvärinen, 1974; Spitzer & Semple, 1993; Warren, Zielinski, Green, Rauschecker, & Griffiths, 2002), although unambiguous demonstrations of selective motion sensitivity are difficult to achieve (McAlpine, Jiang, Shackleton, & Palmer, 2000; Smith, Okada, Saberi, & Hickok, 2004).

**Figure 1 fig1:**
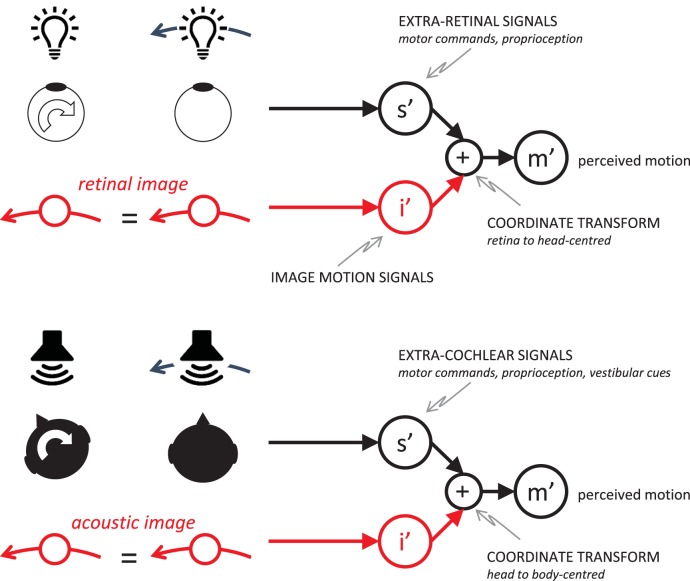
Retinal image motion (top left) and acoustic image motion (bottom left) are both ambiguous—for example, self-movement in front of a static stimulus produces the same image motion as a stimulus that rotates around the perceiver with eye and head still. In vision, this type of problem and its solution are well documented (top right). Here we investigate whether the auditory system takes into account head rotation by using “extracochlear” signals (*s*′) that encode head movement. Combining these with acoustic image motion signals (*i*′) effects a coordinate transform, producing an estimate of movement with respect to the body (*m*′). See the online article for the color version of this figure.

Critically, head movement causes motion in the acoustic image in the same way that eye movements cause motion in the retinal image. A simple example is head rotation in front of a static sound. As schematized in Figure 1, the resulting acoustic image motion is identical to the motion created by a sound source moving in an arc around the listener (note that the Doppler shifts or intensity changes created by the translation of the ears toward and away from the sound source in this example are negligible for reasonable speeds of rotation). Like vision, therefore, hearing faces a similar yet fundamental problem—how to avoid confusing acoustic image motion created by self-movement with the actual movement of sounds. One possible solution is to incorporate “extracochlear” estimates of head velocity, based on a mixture of motor commands, proprioception, and vestibular information, as shown on the right of Figure 1. Simply knowing the head is moving is enough to decide that the (identical) acoustic motion information associated with the two situations in the bottom-left of the figure must arise from sound sources that are moving differently. Yost, Zhong, and Najam (2015) found that listeners were able to make these types of simple categorical judgments during whole-body rotation, albeit with acoustic image motion displayed at very low temporal resolution (repeated 200-ms bursts of stationary noise that were shifted in position during 133.3 ms of silence). Listeners are also able to make use of information about the direction of head rotation, as shown by the reduction of front-back confusions that occur when trying to localize narrow-band stimuli during head movement (Brimijoin & Akeroyd, 2012; Kim, Barnett-Cowan, & Macpherson, 2013; Perrett & Noble, 1997a, 1997b; Wightman & Kistler, 1999). In addition, head movements help promote sound-source externalization (Brimijoin, Boyd, & Akeroyd, 2013) and improve sensitivity to moving sources (Brimijoin & Akeroyd, 2014b). But if the auditory system had access to extracochlear estimates of head *velocity,* then adding these to acoustic motion signals would allow a more generally useful perceptual representation because it implements a coordinate transform. As shown on the right of Figure 1, acoustic image motion would be converted into body-centered movement, just as extraretinal eye-velocity signals in vision convert retinal motion into head-centered movement. This would be one step in the process that prevented the auditory system from mistaking head-centered image motion for real motion and solving ambiguities like those shown in the figure.[Fig-anchor fig1]

**Figure 2 fig2:**
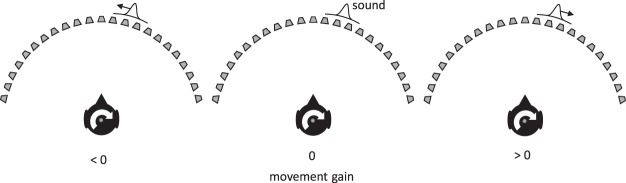
A moving sound source was produced by displacing a Gaussian envelope across an arc of speakers, each producing independent broadband noise. The velocity of the envelope was linked to real-time measurements of the head movement. The movement gain (*g*) defines the dependency: when *g* < 0 (left panel), the sound appeared to move in the opposite direction to the head; when *g* > 0 (right panel), the sound moved with the head; when *g* = 0 (middle panel), the sound did not move at all. The proportion of the measured head speed used to move to the source was set by the value of the *g.*

Our first experiment therefore examined how well listeners are able to convert head-centered acoustic image motion into body-centered movement during head rotation. To do this, we instructed participants to judge whether a sound moving across a horizontal arc of speakers appeared to be moving with or against a self-initiated head rotation (see Figure 2). They were instructed to do this in world-centered coordinates, judging the motion as if it were moving across the arc of speakers (note that because listeners moved only their heads, world-centered and body-centered coordinates coincide). Crucially, sound-source motion was made contingent on the real-time measurement of the head movement, such that its speed across the speakers was a specific proportion of the head’s rotational velocity. We term the proportion the *movement gain* and for simplicity allow it to be signed so as to define source motion with respect to the head movement (see Figure 2). When the movement gain was positive, the sound moved in the same direction as the head; when negative, it moved in the opposite direction; and when 0, it did not move at all. By using different motion gains across a set of trials, we were able to use standard psychophysical procedures to determine the movement gain that made the sound appear stationary with respect to the world/body. We predicted that if the auditory system was able to compensate for head rotation perfectly, the movement gain at this “point of subjective equality” (PSE) should be equal to 0. Conversely, if there was a complete failure to compensate, the PSE should be equal to 1 (the sound would move exactly with the head). Values lying somewhere in between would represent compensation for head rotation that was present but incomplete.[Fig-anchor fig2]

## Experiment 1: Auditory Compensation for Head Rotation as a Function of Head Speed

We investigated head movement compensation for three target head speeds: 20°/s, 60°/s, and 100°/s. Given that head movements were self-controlled, we ran training phases prior to data collection to help participants execute smooth head movements that approximated the target values. Training phases were also interspersed during the testing phase to help maintain the desired target head speed. Testing was carried out in the light but with eyes closed. The room illumination allowed the experimenter to visually monitor head movements in the testing phase.

### Method

#### Stimuli

Sounds consisted of independent broadband noises presented at an overall level of 72 dB(A) across an array of 23 speakers (Cambridge Minx satellite speakers), spaced 7.5° apart, as shown in Figure 2. Sound levels were measured with a handheld sound-level meter at the position of the listener’s head. The speaker array extended ±82.5° from the center, and listeners were positioned so that they faced the central speaker. The physical diameter of the array was 1.4 m. Sounds were controlled by MatLab and delivered by a 24-channel D/A converter (MOTO 24i) at a sampling rate of 48 kHz. A sound position was created by applying a Gaussian-shaped gain function, the source envelope, across the speaker array. The standard deviation of this envelope was 3.75° (half the speaker spacing), large enough to avoid a modulation in level as the envelope moved between speakers but small enough to give the impression of a reasonably punctate and localizable sound source (this is a form of cross-fading, but in which three or more speakers can be active at any one time rather than just two). In the training phase of the experiment, the movements of the envelope were determined by the desired head velocity. In the testing phase, however, the position of the envelope (μ) at time sample *t* was determined by the current head velocity (*V*_*t*_), such that $\mu_{t}=\mu_{t-1}+mV_{t}\delta t$, where *m* is the movement gain and δ*t* is the reciprocal of the sampling rate (see below).

Head movements were measured using a magnetic motion tracker (Polhemus Liberty) consisting of a small sensor worn on a headband, coupled with an electromagnetic source positioned close by. The system was boresighted at the start of each testing session by having listeners position their head so that their nose pointed comfortably at an amplitude-modulated broadband noise being emitted from the speaker at the center of the array. The motion tracker sampled head position at a rate of 240 Hz, which consequently set the temporal resolution of the moving sound source in the testing phase. The same temporal resolution was used in the training phase. We measured the initial delay of the whole system to be around 40 ms (i.e., the time between measuring the first head rotation and delivering a sound). Given that the experiments reported here concern source motion and movement gain, this small fixed temporal offset is inconsequential. Nevertheless, sound intensity was ramped on linearly over the first 500 ms of each trial.

The start location of the envelope was drawn randomly on each trial from the range ±7.5° from straight ahead. Sound-source intensity was gated so that listeners only ever heard the stimulus when their head was moving. To achieve this, head speed was calculated using the difference between the current sample and the one before. The result was then fed through a leaky integrator that helped smooth out small bursts of sound that can arise when differentiating noisy measurements from the motion tracker.

#### Procedure

The three head speeds were investigated in separate sessions, each comprising a training phase and a testing phase.

##### Training phase

Listeners were presented target sounds that moved back and forth at a set speed and asked to track them as accurately as possible with their nose. Each target sound sequence lasted for 12 s and moved back and forth at a constant speed across an arc extending ±30° from straight ahead. Individual training sweeps therefore lasted for 3, 1, or 0.6 s for the target speeds of 20°/s, 60°/s, and 100°/s, respectively. This yielded 4, 12, or 20 sweeps per sequence. To aid training, visible markers were placed on the leftmost and rightmost speaker that marked the ends of the 60° excursion distance. Plots of measured head movement against desired speed were reviewed by the experimenter. If any gross head-tracking errors were observed, the training session was repeated.

##### Testing phase

In the testing phase, listeners were asked to reproduce a given trained head speed with their eyes closed while judging whether the sound source appeared to move with or against the head. Trial duration was unlimited; participants made as many head turns as they needed in order to judge the motion. We did not counterbalance start direction, and participants were allowed to move seamlessly to the next trial without stopping their head movement if they so wished. In these cases, the sound source would momentarily be silenced and then intensity ramped up as described above.

A method of constant stimuli was used to collect psychometric functions for each of the three target head speeds investigated. This consisted of presenting six movement gains over the range −0.5 to +0.75 in 0.25 steps; the asymmetry about 0 reflects pilot observations that the compensation was incomplete and centered on a movement gain of about +0.1. Additional pilot work was carried out in Glasgow using different, but functionally equivalent, apparatus and found similar results (Brimijoin & Akeroyd, 2014a). Five replications of each movement gain were presented in a randomized order.

Training and testing phases were repeated four times, yielding 20 replications of each movement gain per target head speed. Each psychometric function was therefore based on 120 trials, and each participant carried out 360 trials in total.

#### Analysis

##### Psychophysics

**Figure 3 fig3:**
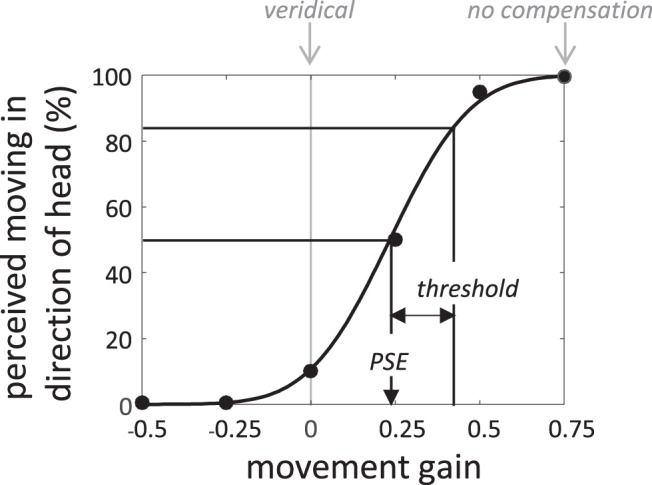
An example psychometric function obtained from presenting a range of movements gains over a series of trials. The ability to compensate for head motion is determined by the point of subjective equality (PSE), which defines the point at which the source appeared stationary to the listener. In this example, the PSE is small and positive, indicating a good but not perfect ability to compensate. The slope of the psychometric function indicates the precision of movement judgments within our setup. We define precision as the difference in movement gain between 84.1% and the PSE.

For each participant and trained head speed, a cumulative Gaussian was fit to the data using probit analysis (an example is shown in Figure 3). The PSE is then defined as the movement gain at the 50% point on the resulting psychometric function. This corresponds to the movement gain where the sound appeared to move neither in the same direction nor the opposite direction to the head rotation—in other words, a perceptually stationary source with respect to the world/body. We also obtained a measure of precision (i.e., a discrimination threshold) using the standard deviation of the best-fitting cumulative Gaussian. The standard deviation corresponds to the difference between the movement gains at 84.1% and 50% (see Figure 3). The threshold therefore provides an estimate of the slope of the psychometric function.[Fig-anchor fig3]

##### Head movements

For each trial, head position samples were first smoothed using a Gaussian filter (*SD* = 8 Hz) and then converted to velocity by taking a time derivative. The root mean square (RMS) speed was then calculated. Accuracy was subsequently determined by calculating the mean RMS speed (collapsed across movement gain) across listeners at each trained head speed.

#### Participants

Thirteen listeners participated for course credit as part of the requirement for their undergraduate studies at Cardiff University. The PSEs for all listeners were positive except for one, who produced a large and negative value at the highest trained speed, 10 *SD*s below the mean. We therefore dropped this person’s whole data set from further analysis, leaving *N* = 12.

### Results

**Figure 4 fig4:**
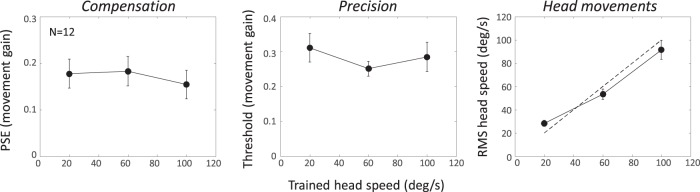
The left panel shows the mean ability to compensate as a function of the trained head speed for 12 listeners. The movement gain is small and positive, indicating good if incomplete compensation. Hence, a stationary sound appears to move slightly against the head movement (in vision, this is called the Filehne illusion); to cancel the perceived motion requires a small amount of source movement in the same direction as the head rotation. The middle panel shows the mean threshold or slope of the psychometric function. The results show that precision of compensation does not vary across a wide range of head speeds. The right panel plots the mean root mean square head speed obtained in the main experiment, with the dotted line indicating ideal reproduction of the trained head speeds. All error bars are ±1 *SE.* PSE = point of subjective equality.

Figure 4 (left panel) plots the mean movement gain at the PSE for the three trained head speeds. The average value is a little over +0.15, indicating that our listeners were able to compensate for about 85% of the speed of the head rotation. Complete compensation would produce a PSE of 0, but all three means were significantly different from 0 (*t*_11_ = 5.70, 5.78, and 5.03, respectively; all *p* < .001). Moreover, there was no effect of trained head-speed condition (*F* < 1).[Fig-anchor fig4]

Figure 4 (middle panel) shows the mean precision across observers. Again, there is no effect of trained head speed (*F*_2, 22_ = 1.50, *p* = .25). Because precision is expressed proportionally with respect to head speed (i.e., the thresholds are in movement gain units), this shows that the threshold for discriminating one movement gain from another is a fixed proportion of the self-movement. The precision data therefore suggest a type of Weber’s law for discriminating sound source movement in our setup. If the thresholds were expressed in °/s, they would therefore increase proportionally with trained head speed, suggesting that one or more of the underlying motion signals used to make the judgment becomes less reliable as average head speed increases.

Figure 4 (right panel) shows the accuracy of the actual head movement during the testing phase, the dotted line indicating ideal reproduction of the trained speed. Recall that sound-source motion was made contingent on head speed in the testing phase. Hence, the measured accuracy does not affect any of the above conclusions; it simply shows the range of head speeds over which they apply. For the slowest speed, listeners moved their heads slightly faster than required, while at the faster speeds, head movements were slightly lower. Hence, the range of speeds achieved in the testing phase was slightly less than desired but still spanned a wide range. If it is assumed that the ability to reproduce the desired head speeds during testing reflects the ability to track a moving sound during training, then our head movement data support the recent findings of Leung et al. (2015).

Our results suggest that the auditory system is able to compensate for head rotation reasonably well. Sounds had to move roughly 15% of the speed of ongoing head movement and in the same direction in order to appear stationary to the listener. This demonstrates that a stationary sound source appears to move slightly against the head rotation. Our findings therefore reveal the auditory equivalent of the Filehne illusion (Filehne, 1922), in which static visible objects appear to move in the opposite direction to a pursuit eye movement (Freeman, 2001; Freeman & Banks, 1998; Mack & Herman, 1973, 1978; Wertheim, 1994).

As detailed in the Discussion, there has been a longstanding debate in vision science as to why perceptual errors like the Filehne illusion occur during self-movement (see Freeman et al., 2010; Furman & Gur, 2012; Wertheim, 1994). At the core of this debate is the idea that the mechanism estimating self-motion produces a lower estimate of speed than the mechanism estimating image motion. Our data therefore imply that a similar speed mismatch operates for the auditory system. In Experiment 2, we sought to generalize this finding by investigating compensation as a function of horizontal eccentricity (azimuth). The ability to discriminate location and motion declines when sounds are placed more eccentrically along the azimuthal plane, with poorest performance obtained when stimuli are in line with the interaural axis (Chandler & Grantham, 1992; Grantham, 1986; Mills, 1958; Perrott & Saberi, 1990; Strybel, Manligas, & Perrott, 1992). We therefore predicted that the ability to compensate for head rotation should also depend on eccentricity.

## Experiment 2: Auditory Compensation for Head Rotation as a Function of Eccentricity

### Method

#### Stimuli and procedure

**Figure 5 fig5:**
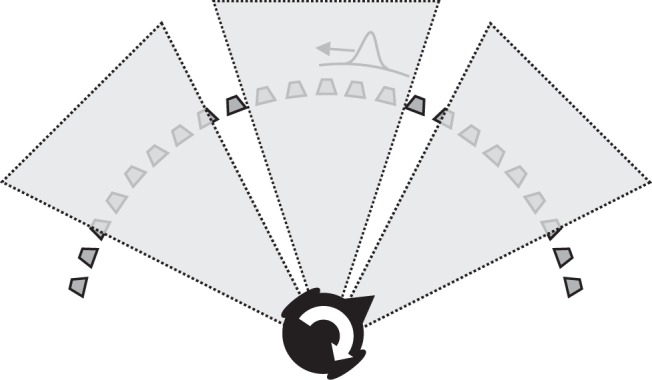
To investigate source eccentricity (azimuthal position), we restricted the sounds to be audible only in the predefined shaded regions shown. Each of these spanned 40° about a mean azimuth of 0°, 45°, or 90° with respect to body centered straight ahead. Because our arc of speakers did not completely surround the listener, she or he was positioned facing the speaker at 45° as shown to achieve the desired eccentricities.

Eccentricities of 0°, 45°, and 90° were investigated using the same moving sources used in Experiment 1 but presented through a software-generated window that constrained where the sound was audible (shaded areas in Figure 5). The initial position of the sound source was at the eccentricity under test. The position of the window was fixed to the speaker array and spanned ±20° horizontally about the desired eccentricity. When the sound moved outside the window, its intensity was set to 0; within the window, the sound was only audible when the head was in motion, as in Experiment 1. Given that our speaker array did not span a full circle, we had to position the listener so he or she faced the speaker positioned 45° right of center as shown in the figure. Hence, it was this speaker on which the motion tracker was boresighted at the start of testing. The eccentricities of 45° (middle shaded area) and 90° (left-hand shaded area) were therefore always situated to the listener’s left.[Fig-anchor fig5]

Psychometric functions were based on nine movement gains ranging from −0.4 to +0.4 in 0.1 steps. Each listener completed two sessions of 90 trials at each eccentricity, yielding 20 trials per gain value. Prior to data collection, a few practice trials were given so that listeners could familiarize themselves with the procedure. Unlike Experiment 1, however, there was no explicit head-speed training—listeners were simply told to move their heads smoothly, horizontally, and back and forth. PSEs, thresholds, and head movements were analyzed in the same way as Experiment 1.

#### Participants

Twelve new listeners participated for course credit as part of the requirement for their undergraduate studies at Cardiff University. One listener produced a threshold over 10 *SD*s above the mean so this person’s whole data set was dropped from further analysis, leaving *N* = 11.

### Results

**Figure 6 fig6:**
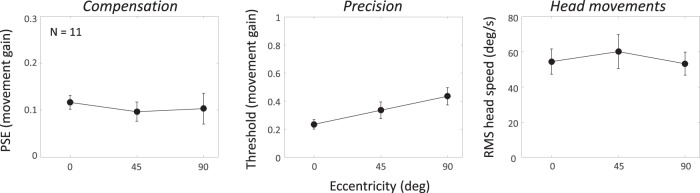
The left panel shows the mean ability to compensate as a function of eccentricity (azimuth) for 11 listeners. The results replicate the small but positive movement gain found in Experiment 1 and show that compensation is independent of horizontal position. The middle panel shows the mean threshold and the right panel the mean root mean square (RMS) head speed. All error bars are ± 1 *SE.* PSE = point of subjective equality.

Figure 6 (left panel) shows the mean PSEs across listeners. There was no effect of eccentricity (*F* < 1), with each mean significantly above 0 (*t*_10_ = 7.82, *p* < .001; *t*_10_ = 4.7, *p* < .001; and *t*_10_ = 3.12, *p* = .01, respectively). The threshold data shown in the middle panel suggest that precision declined with eccentricity (*F*_2, 20_ = 6.12, *p* = .008), even after correction for a violation of sphericity (*p* = .02). The head movement data shown in the right panel suggest that the change in precision was most likely due to those mechanisms estimating acoustic image motion because the mean RMS head speed—a proxy for the “size” of the extracochlear signal—did not vary (*F*_2, 20_ = 1.69, *p* = .21). As in Experiment 1, therefore, we found that the auditory system is able to compensate for head rotation reasonably well, with the small error indicating the stationary sounds appear to move slightly against the direction of head movement. This error was numerically slightly smaller than in Experiment 1, but on closer inspection, the two most comparable conditions (straight ahead vs. medium trained head speed) were not significantly different (between-subjects *t*_21_ = 1.89, *p* = .07). Contrary to expectations, however, compensation did not depend on eccentricity despite the change in precision we found.[Fig-anchor fig6]

## General Discussion

We used a technique that presented sounds to a listener contingent on the velocity of his or her head movement and found that the auditory system is able to compensate for the motion of the sensory apparatus, although not completely. In two experiments, we found a consistent if small perceptual error: Listeners hear a stationary sound as moving slightly opposite to the direction of an ongoing head rotation, and this persists across a wide range of head speeds and azimuthal locations. The perceptual error is the auditory equivalent of the Filehne illusion, a motion phenomenon associated with the apparent movement of stationary visual objects during smooth pursuit eye movement (Freeman, 2001; Freeman & Banks, 1998; Mack & Herman, 1973, 1978; Wertheim, 1994).

The visual Filehne illusion, along with other pursuit-based phenomena, is taken as evidence that the visual system attempts to convert retinal motion into a coordinate system centered on the head but that this conversion is incomplete (Furman & Gur, 2012). Our experiments therefore provide evidence that the auditory system attempts a similar coordinate transform, this time from head centered to body centered, but again, the conversion is incomplete. To achieve this, the auditory system needs information to estimate the velocity of the head, which in the context of our experiments must come from a source other than the acoustic image. We refer to this as “extracochlear,” in keeping with the definition of “extraretinal” signals known to accompany smooth pursuit eye movements in vision.

The fact that both sensory modalities produce this form of perceptual error is surprising. Vision, for instance, has ample opportunity to interrogate eye-movement accuracy and retinal and extraretinal motion signals each time a pursuit eye movement is performed. In vision, therefore, one might expect the sort of perceptual errors defined by the Filehne illusion to be calibrated out (Bompas, Powell, & Sumner, 2013; Freeman et al., 2010; Valsecchi & Gegenfurtner, 2016). Given that they are not, one might expect similar explanations for the existence of these persistent errors for both vision and hearing. Here, we briefly review two possibilities put forward for vision and ask to what degree they might account for the type of error we have found in hearing.

The first “mechanical” type of explanation argues that perceptual mistakes arise from fundamental and immutable errors in the early sensory apparatus. Thus, in the case of the Filehne illusion and other associated phenomena, the initial measurements made by retinal motion signals are higher in value for a given speed compared to extraretinal motion signals (Freeman, 2001; Freeman & Banks, 1998; Wertheim, 1994). To see why, consider pursuit at velocity *P* over a stationary object. This produces an equal and opposite image velocity of –*P.* From Figure 1, perceived motion *m*′ = *s*′ + *i*′, where *s*′ is the estimate of eye pursuit and *i*′ the image motion. If we assign *e* and *r* to define the respective gains of the initial speed measurements, then *m*′ = (*e* – *r*)*P.* Hence, when extraretinal signals register a lower speed (*e* < *r*), a static object will appear to move against the eye movement (i.e., *m*′ < 0). This is the Filehne illusion. Exactly the same argument can be made for the auditory system, this time based on potential differences in speed estimates made by acoustic and extracochlear signals.

As suggested above, however, the fact that these initial measurement errors are not calibrated out is puzzling. The second type of explanation, based on Bayesian statistics, perhaps points to a more principled reason for why perceptual errors like the Filehne illusion occur (Ernst & Bülthoff, 2004; Weiss, Simoncelli, & Adelson, 2002). In a nutshell, accuracy is sacrificed for precision. The Bayesian explanation acknowledges that incoming sensory evidence is imprecise for a variety of reasons, some external to the perceiver and due to context (e.g., stimulus contrast, background noise) and some internal to the observers and due to neural noise; perception is therefore about making an optimal decision or “best guess” in the face of unreliable evidence. To achieve this, imprecise sensory evidence (represented by a likelihood function) is multiplied by a prior distribution defining the perceiver’s expectations about the world. This yields a posterior distribution, the average of which defines (in the case of motion) perceived speed. For vision, the prior expectation is that most objects are stationary or move slowly (Weiss et al., 2002). Hence, as the reliability of sensory evidence declines, such as occurs when stimulus contrast is lowered or a target is pursued, the posterior moves toward the prior and the estimate of speed reduces. Note that the sensory measurements captured by the likelihood function are often assumed to be accurate on average—that is, the observer is assumed to be *unbiased* (Alais & Burr, 2004; Ernst & Banks, 2002; Freeman et al., 2010). Hence, for the visual Filehne illusion, extraretinal signals produce lower estimates of speed, not because the initial measurements are less accurate, as the mechanical explanation argues, but because they are less precise and so more dominated by the prior.

The mechanical explanation could certainly explain the auditory Filehne illusion we describe here—acoustic image motion signals could, for some reason, provide higher measurements of speed than extracochlear signals. But a Bayesian explanation may also account for the data and place it within a broader explanatory framework. For instance, recent evidence has looked at the auditory equivalent of the effect of stimulus contrast on perceived speed, a phenomenon that has been at the heart of the debate over the degree to which vision behaves in a Bayes-like manner (Hassan & Hammett, 2015; Hürlimann, Kiper, & Carandini, 2002; Sotiropoulos, Seitz, & Seriès, 2014; Stocker & Simoncelli, 2006). Lowering contrast lowers the reliability of the sensory evidence; hence, the posterior shifts toward the slow-motion prior and reduced perceived speed. With this in mind, Senna, Parise, and Ernst (2015) showed that making an acoustic signal less reliable by presenting it within spatially diffuse broadband noise also makes the sound appear to move more slowly. They accounted for the perceived slowing by appealing to the idea that hearing shares the same or a similar slow-motion prior to vision. This idea has been extended to account for a tactile version of the Filehne illusion (Moscatelli, Hayward, Wexler, & Ernst, 2015). The fact that vision, audition, and touch produce similar perceptual errors suggests there may be a common underlying mechanism such as that described by the Bayesian framework.

In Experiment 2, however, we failed to find a change in the accuracy of compensation at different eccentricities, despite a change in precision. On the face of it, therefore, the findings of Experiment 2 do not support a Bayesian explanation of the auditory Filehne illusion. However, there are at least two reasons why such a conclusion may be premature. First, the inference is based on averages across listeners, but quantitative Bayesian models are typically tested against individual data because group-level statistics fail to account for individual differences in sensitivity and priors (Adams, 2007; Edden, Muthukumaraswamy, Freeman, & Singh, 2009; Freeman et al., 2010; Hibbard, Bradshaw, Langley, & Rogers, 2002; Nefs, O’Hare, & Harris, 2010; Song, Schwarzkopf, Kanai, & Rees, 2015; Sotiropoulos et al., 2014; Stocker & Simoncelli, 2006). Without knowing how priors vary, it is difficult to predict the degree of compensation one might expect based on thresholds. The second reason is that our thresholds combined two sources of noise, one extracochlear and one acoustic. At present, it is difficult to know how to separate these out, but this would need to be done in order to predict the degree of change in compensation one might expect (Freeman et al., 2010). As they stand, the thresholds therefore contain no information about the relative size of the two underlying noise sources. This is problematic because in some individuals, the level of extracochlear noise may have been relatively high, swamping any location-dependent changes in threshold driven by the acoustic signal. In others, the level may have been relatively low, thus revealing these changes. One potentially fruitful line of enquiry would be to impose larger changes in acoustic noise by external means. Unlike vision, however, the study of stimulus reliability and its effect on perceived auditory speed has only just begun (Senna et al., 2015).

In summary, we find that the auditory system is able to compensate for head rotations during the perception of sound-source motion but, like vision, the compensation is incomplete. The fact that these perceptual errors exist should prove a rich arena for further exploring coordinate transforms in the auditory system, as well as commonalities in motion processing across the senses.
